# Clustering and mapping the first COVID-19 outbreak in France

**DOI:** 10.1186/s12889-022-13537-7

**Published:** 2022-07-01

**Authors:** Regis Darques, Julie Trottier, Raphael Gaudin, Nassim Ait-Mouheb

**Affiliations:** 1 UMR 7300 ESPACE, CNRS, Aix Marseille Univ, Université Côte d’Azur, Avignon Université, Case 41, 74 rue Louis Pasteur, 84029 Avignon cedex, France; 2 CNRS, PRODIG, Campus Condorcet, Bat. Recherche Sud, 5 cours des Humanités, 12 rue des Fillettes, 93322 Aubervilliers cedex, France; 3 Institut de Recherche en Infectiologie de Montpellier (IRIM), CNRS, Univ Montpellier, 1919 Route de Mende, 34293 Montpellier, France; 4 UMR G-Eau, INRAE, University of Montpellier, 361 rue Jean-François Breton, 34196 Montpellier cedex 5, France

**Keywords:** France, COVID-19, SARS-CoV-2, Spatial clustering, Epidemiology, Background

## Abstract

**Background:**

With more than 160 000 confirmed COVID-19 cases and about 30 000 deceased people at the end of June 2020, France was one of the countries most affected by the coronavirus crisis worldwide. We aim to assess the efficiency of global lockdown policy in limiting spatial contamination through an in-depth reanalysis of spatial statistics in France during the first lockdown and immediate post-lockdown phases.

**Methods:**

To reach that goal, we use an integrated approach at the crossroads of geography, spatial epidemiology, and public health science. To eliminate any ambiguity relevant to the scope of the study, attention focused at first on data quality assessment. The data used originate from official databases (Santé Publique France) and the analysis is performed at a departmental level. We then developed spatial autocorrelation analysis, thematic mapping, hot spot analysis, and multivariate clustering.

**Results:**

We observe the extreme heterogeneity of local situations and demonstrate that clustering and intensity are decorrelated indicators. Thematic mapping allows us to identify five “ghost” clusters, whereas hot spot analysis detects two positive and two negative clusters. Our re-evaluation also highlights that spatial dissemination follows a twofold logic, zonal contiguity and linear development, thus determining a “metastatic” propagation pattern.

**Conclusions:**

One of the most problematic issues about COVID-19 management by the authorities is the limited capacity to identify hot spots. Clustering of epidemic events is often biased because of inappropriate data quality assessment and algorithms eliminating statistical-spatial outliers. Enhanced detection techniques allow for a better identification of hot and cold spots, which may lead to more effective political decisions during epidemic outbreaks.

**Graphical Abstract:**

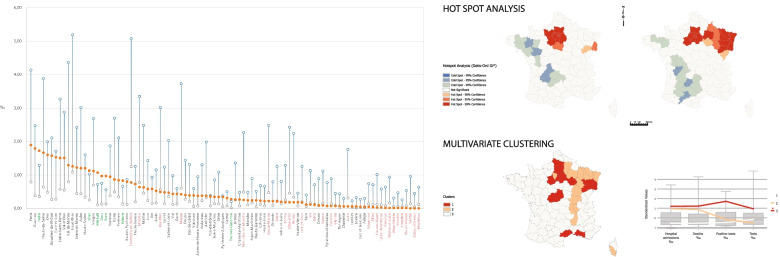

## Background

With the worldwide expansion of COVID-19 in the spring of 2020, people were urged to rethink their relationship with space and their everyday environment. Social distancing, isolation, quarantine and community containment created an infinity of invisible barriers that might be considered as a general “rebordering” process affecting space and society. Against this, some analysts support the idea that “viruses know no borders” [1, 2], meaning that travel bans and fences are inappropriate measures for attaining the objective of protecting people against a respiratory virus.

The legitimacy and acceptability of quarantine and self-protection initiatives is widely discussed in the literature today, especially since the 2000s and SARS-related experiences, with mitigated and contradictory conclusions [3–6]. Mass quarantine is often regarded as a controversial measure, but the isolation and treatment of symptomatic individuals are expected to be more effective responses to emerging epidemics.

In fact, isolation strategies imply partitioning space in a variety of ways. The operation is as challenging as preventing the spread of germs, mainly because human societies are built on proliferating intertwined relationships taking the form of contacts, flows, and exchanges [7]. In social terms, boundaries tend to depress interactions to such a point that forced immobility, limited exchanges, and deficient communication are signs often associated with decay or even death [8].

These considerations lead us to the question of how the coronavirus crisis has been managed in epidemiological and geographical terms [9]. Dozens of countries worldwide have been *forced* to stringent lockdown decisions, be they limited to cities, counties, regions, or even entire states. The variety of answers adopted by national or local authorities diversely impacted by the pandemic produced complex situations: regions and population groups affected by similar levels of contamination could be constrained by severe movement restrictions or, rather the opposite, allowed to move freely. Such seemingly inconsistent decisions derive from the configuration of health systems, diverging legal frameworks, diversely stratified decision-making processes, but also from the inability to identify emerging clusters. Uncertainty in this area produces deleterious effects, such as global “blind” decisions, the effectiveness of which is not guaranteed. This “sideration” effect associated with seemingly inconsistent decisions is sometimes assimilated with chaos or phase transition [10, 11].

France is one of those countries which adopted for a time global lockdown as the best way to *wage war* against the coronavirus (March 17-May 11, 2020) [12]. The conditions for this stringent lockdown were fixed by Decree 2020–293, March 23 [13]. People were effectively placed under house arrest. Outdoor circulation was authorized within a distance of one kilometer from home for a period of one hour a day. All economic and social activities were idled, with the exception of essential services such as health care, energy and supply trade, food shops, and all activities open to special arrangements such as remote work (education, bank, insurance, etc.). A number of industrial facilities, warehouses, and wholesale markets were authorized to operate but decisions were taken on a case-by-case basis, which contributed to creating confusion. Breach of the obligation was penalized by a fine. The lockdown was *global*, e.g. the whole country was affected by the anti-epidemic measures without local exception in metropolitan France.

On June 22, the day when primary and secondary schools reopened, the number of diagnosed cases reached 160 000 and the number of deaths was about 30 000. With 454 deaths per million inhabitants, the country stood in sixth position among the most impacted places worldwide -leaving aside microstates such as San Marino and Andorra. This average result conceals a much more complex reality where local extreme situations compensate for areas almost entirely spared by the virus. The national crisis has been acute, and the stringent lockdown strategy adopted by the government has been hotly disputed [5]. Some observers find that the lockdown was very effective in reducing the spread of the disease [14], others think that the price to be paid was really heavy, especially in economic terms.

While most scientific studies focus on assessing the extent of the spread through more or less conventional geo-epidemiological models with the intent to simulate further infection developments -SEIR compartmental models or agent-based models, out of many examples-, we believe that thoroughly re-analyzing official statistics about COVID-19 is the best way to support public health decision-makers. Modeling is an excellent option when the conditions of collection and processing of data are fully specified and mastered, which is not the case with many COVID-19 official databases. When these conditions are not met, modeling -and especially the array of methods used in the area of epidemiology- often produces errors, hence the inability of COVID-19 models to “predict” future disease developments, particularly in spatial terms [15]. To put it otherwise, data quality assessment is a firm condition for studying the epidemic further. A number of studies used heterogeneous data resources erroneously, particularly at an international scale level, which produced inconsistent results despite the introduction of highly efficient algorithms and modeling techniques.

We hypothesize that the debate has been biased in different ways. (1) The attention placed on deaths (mortality, lethality, morbidity, fatality) has left aside other relevant indicators able to address active epidemic propagation [16, 17]. (2) Through their crucial role in managing the crisis and *defeating* the pandemic, health structures and care systems have imposed their own rationale [18, 19]. (3) We assume that the most advanced mathematical models cannot compensate for degraded initial statistics, which often produces mixed results [20, 21]. (4) Scale is a problematic topic. Most epidemiological research follow a micro or macro scale approach, but intermediate levels are neglected. This is a major shortcoming for surveillance authorities whose mission is the pursuit of excellence in care for persons through territorial management.

Against other studies that use the number of deaths and confirmed COVID-19 cases in conjunction with low-quality and “almost random” socio-demographic or environmental covariates -e.g., whose relationship with the epidemic is more suspected than confirmed [16, 22]-, we take as a premise that introducing ancillary data into simulation models might be more effective once spread patterns are better identified. This could break the cycle of powerlessness for national authorities who realize little benefits from macro-scale infectious disease simulation tools or micro-scale interpersonal contamination models.

Our objective is to assess the efficiency of public lockdown policy in limiting spatial contamination through the analysis and use of official statistics in France during the first epidemic outbreak and its immediate aftermath. Considering the organic relationships between health care, national security, and statistical production systems, particular attention will be paid to the understanding of data quality because of frequent misuses of statistics [23, 24]. This study is positioned at the crossroads of geography, geomatics engineering, spatial epidemiology, and health sciences [25].

Early epidemic detection is a key opportunity for the authorities. During the emergence phase of a disease, efficient measures can still be adopted and implemented, not only in the area of health prevention, but also through geographic-based containment decisions. Global lockdowns are last resort solutions; they should be taken as evidence of a failure of prevention policies. During the first COVID-19 outbreak worldwide, few countries managed to contain the spread of the virus. One important reason that led to this pandemic may come from our inability to use geographic-based tools to isolate population at the local level. Indeed, whether this is due to a technologic, conceptual or logistic gap of knowledge, it appears clearly nowadays that addressing the early spread of pathogens across defined areas would be useful before infected populations develop symptoms and eventually be treated in hospital units. This supposes that mass, vs. random, vs. targeted testing be conducted as early as possible among broader sections of the population, be it through direct population testing or indirect environmental detection.

Here, we propose to retrospectively re-analyze the data we had in hands at the time of the first French lockdown using a geographic-based approach, including clustering and mapping processes, to improve preparedness toward future pandemics. Specifically, we studied the spread of the virus using spatial autocorrelation, thematic mapping, hot spot clustering, and multivariate analysis. This work shall be seen as a contribution to the development of common tools, methods and strategies able to improve detection and monitoring systems, so that policy-makers can assess local epidemic status and trigger most appropriate responses. These methods can readily be adapted to virtually any location worldwide.

## Methods

### COVID-19 metrics, indicators, and maps

Selecting the most appropriate statistical-spatial variables supposes at first to get insight into the French statistical system developed under the authority of Santé Publique France (France Public Health) and the French Ministry of Health and Solidarity [26]. Although WHO data collection about COVID-19 started January 11 [27], France begins to develop its own statistical database on March 3, e.g., 14 days before the general lockdown is declared (March 17). The observation system is not immediately operational and gains momentum through a stepwise process. The number of core indicators is very limited and based almost exclusively on data gathered through hospital units or associated services (SOS médecins, biomedical analysis laboratories). Public communication focuses on the number of reported hospital admissions, admissions in intensive care units, and deaths, through the form of an official dashboard associating raw figures, graphs, and maps.

Core information is provided by the ARS (Regional Health Agencies) network based on data from local hospital units and health services. Public dissemination is ensured at the department and region levels, thus reflecting the administrative structure of the ARS [28]. These are agencies created in April 2010 following an important reform of the health care system (PNR, National Reform Program, Law HPST (Hospital, Patients, Health, Territories) dated July 21, 2009). French regional divisions derive from Law NOTRe (New Territorial Organization of the Republic) of August 7, 2015, which granted enhanced competencies to regions encompassing a larger number of departments. The COVID-19 management system derives from these earlier reforms.

**Fig. 1:**
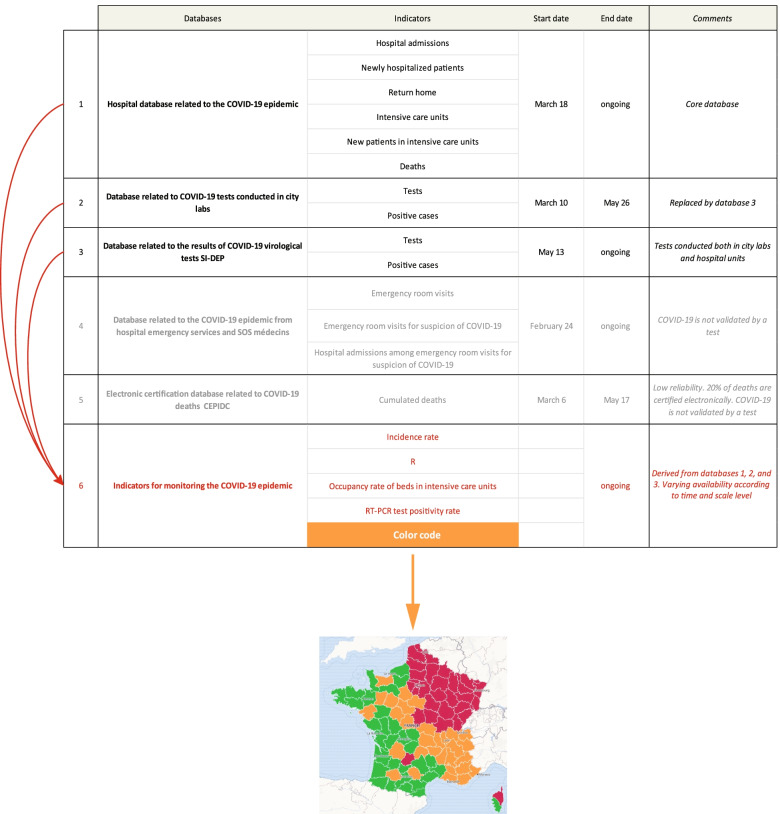
Santé publique France main databases and indicators about the COVID-19 epidemic, and derived vigilance map. Source: Own elaboration based on data from Santé publique France, Etalab. There are five main databases to be used for COVID-19 monitoring. The sixth one derives from previous data 1–3 and supports decision making for the authorities in charge of defining legal requirements and concrete steps to be taken. These derived indicators serve, in turn, as a basis for further mapping through color-coded areas. The process is top-down and straightforward

A “vigilance map” developed by the authorities and color-coded green-orange-red defines alert levels and subsequent lockdown enforcement measures [26] (Fig. 1). Figures and maps are thus used as a justification for further political and administrative decisions whose limits are ultimately defined by the government. Local prefectures whose mission is to maintain law and order and safeguard internal security are granted with enforcement powers. The regional prefects are also chairmen of the Supervisory Board of the ARS.

### Assessing the quality of official statistics

**Fig. 2:**
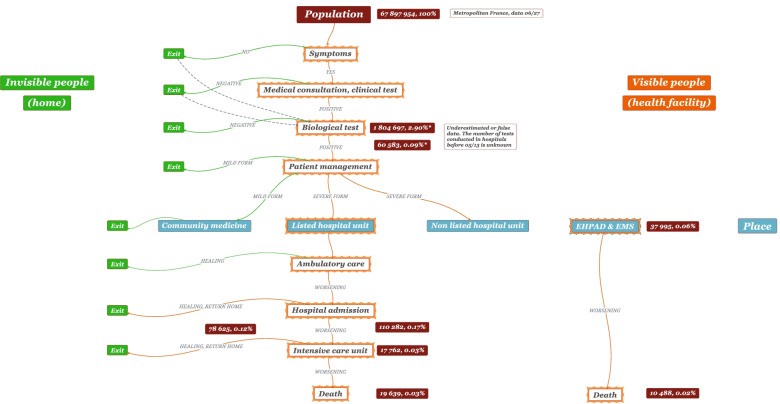
Care pathways, general prevalence. Visible and invisible people from a statistical and geographical perspective. Source: Own elaboration based on data from Santé publique France, INSEE. This flow chart should be read from top to bottom. On the left side and in green are the exits from care pathways. On the right side and in orange are to be found the visible individuals. The blue color introduces the geographical variable and also defines the interface between civil society and health care system. There is no information before testing. The authorities do not provide any further details about community medicine. The lower part of the chart depicts the procedure and arrangements for patient care. Some hospital units involved in the COVID-19 system at different degrees remain out of sight. Despite the number of cases observed, EHPAD and EMS are marginally present

These databases call for various comments about potential uses, limitations, and significance, all the more so as data collection procedures are not detailed by the authorities. This implies for us to evaluate the relevance and usefulness of statistical information ex-post:

1. In the absence of any information at a sub-departmental level, many geographical analyses are simply impossible to carry out. Unlike other countries, there is for instance no way to address urban vs. rural discrepancies as regards the virus spread in France [29]. More refined data exist, but they are not released publicly. Public information mainly comes from the network of hospital units belonging to the official list of frontline medical centers, or from the list of biomedical city labs. Such a limitation is even starker for intra-urban analysis.
2. This fundamental lack of data on the issue produces a side effect. The official geography of COVID-19 is entirely driven by administrative vector features. The coronavirus seemingly shows respect for departmental and regional boundaries whose demarcations constrain dissemination patterns. The vigilance map elaborated by Santé publique France is a primary example of how the government and public administrations have the upper hand on the epidemic [30]. The switch from one color-coded classification to the next one is most often done at a regional level, which means that the epidemic proceeds bureaucratically.
3. Surprisingly, community/city medicine is evacuated from the COVID-19 observation system. The only exception is database 2, replaced by database 3 after May 13 (Fig. 1). Everything seems to happen inside the walls of a limited number of hospitals. Database 4 is the only one relating sick population with hospital admissions. The consequences of this situation are manifold and profound. The coronavirus is apprehended almost exclusively through the lens of critical pathological conditions requiring technical targeted therapy that cannot be achieved outside specialized hospital units. Asymptomatic people, infected individuals on home care -be they tested or not-, and low-risk populations remain hidden in the background.
4. EHPAD (residential care facilities for dependent elderly persons) and EMS (medical-social health-care institutions) counting a large number of casualties have been left aside up to April 1 because of their specific position within the national health care system.
5. To better understand what is at stake with these databases, it should be noted that the authorities take great care to avoid the term *prevalence*. Santé publique France produces two important indicators: (a) the *incidence rate*, otherwise called *epidemic activity*, is defined as the number of positive tests per 100.000 inhabitants over one week; (b) the RT-PCR test positivity rate is defined as the number of positive tests as a percentage of the number of tests conducted over one week. Prevalence defined as the number of infected people within the general population is never calculated. Given that a very small number of tests have been conducted in France, prevalence as the only concept able to assess the global spread of the virus remains out of reach. In other words, the tested sample is so small that it should never be considered representative both in statistical and geographical terms. We have observed repeated confusion as regards these concepts, including in international databases [31–33].
6. To sum up, just like in other countries where integrated care pathways have been developed, the entire observation system is based on monitoring the epidemic through the management of patients and among them the ones suffering from the worst COVID-19 forms. Figure 2 shows how much this process is narrow in scope and reductionist in nature both in statistical and spatial terms.

### Addressing COVID-19 statistically

Given these constraints on statistical data, our attention will focus on three databases:

- Database 1Hospital database (March 19-June 27) relating to the COVID-19 epidemic. This provides information about the COVID-19 situation in referenced hospital units. Data are supplied at departmental and regional levels. There are four available variables: (1) daily number of newly hospitalized persons; (2) daily number of new intensive care admissions; (3) daily number of newly deceased persons; and (4) daily number of new home returns. Our attention focused on hospitalized persons and deceased patients.
- Database 2 Diagnostic tests conducted in city laboratories (March 10-May 26). There are two variables: total number of tests and positive tests. Data are supplied at a departmental level. The *3labos* surveillance system used an automated data transmission process conducted by three laboratories in charge of sample centralization (Eurofins, Biomnis, and Cerba). The system was operational during the general lockdown. Update was stopped on May 29 2020 for unexplained reasons.
- Database 3Results of COVID-19 virological tests SI-DEP (May 13-June 27). The SI-DEP database is presented as a more finalized version of database 2 although data collection takes a different form. Tests conducted in city laboratories and hospital units are mixed. Thus, there is no continuity with database 2. The system is still operational today.

For a full description of the above, see the corresponding web pages of Santé publique France (see section Availability of data and materials).

These datasets were downloaded from the governmental online platform on June 30 2020. Most primary indicators show drawbacks implying different ex-post rectifications. These are conducted by Santé publique France. By definition, registered patients were tested positive, which motivated their inscription into the databases. The actual cause of death reported in database 1 is a much-disputed issue because of potentially associated comorbidities. This has given rise to the controversy about deaths “reported with COVID-19” or “caused by COVID-19”.

The idea is to mirror hospital metrics with the closest approximation to a prevalence indicator, e.g., test results. The advantage of database 2 is double: it allows focusing on community medicine, with an all-embracing dimension on civil society, and it provides the best proxy for general prevalence. Unfortunately, this database is suppressed on May 26.

**Fig. 3:**
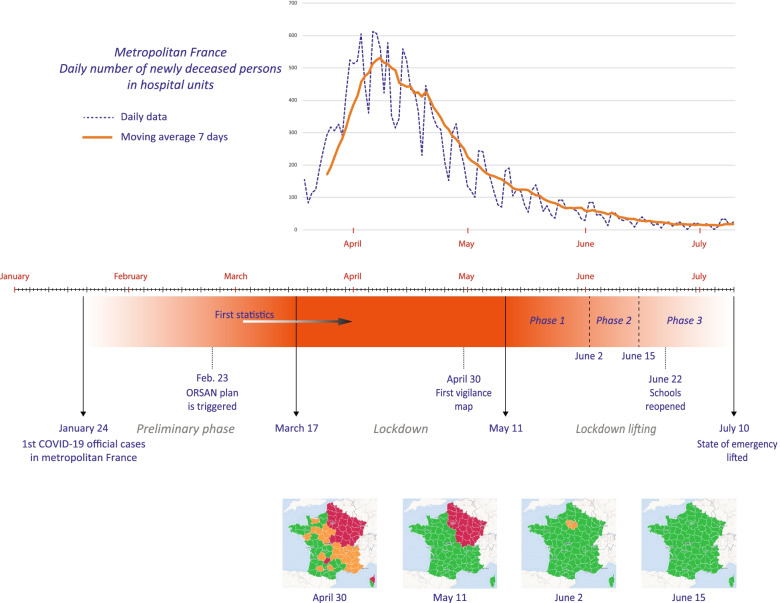
Brief chronology of COVID-19-related events in France (January-July 2020, first epidemic outbreak). Source: Own elaboration based on data from Santé publique France

Timing is determined both by data availability and the chronology of events. Figure 3 provides an overview of the COVID-19 legal management process. The analysis is completed once the most important movement restraints are lifted at the end of June with schools reopening and, simultaneously, the beginning of summer holidays and people regaining mobility. All cards are then reshuffled. July 10 might be seen as a symbolic-legal date. The March 10-May 26 period (database 2) is thus crucial because it covers the worst phase of the epidemic. At both ends of the curve daily deaths in hospitals are about one hundred.

We intend to map the epidemic at a sub-national level. The scale adopted must always be as accurate as possible (department). The solution to be favored will be to map the closest approximation to a prevalence indicator (tests and positive tests), voluntarily leaving aside the management of patients within hospital units. For an enhanced clarity and comparability of results, all indicators will refer to the local population estimates (2020) provided by the INSEE (National Institute of Statistics and Economic Studies). Besides, we assume that composite, standardized indicators are not the most appropriate variables to consider with databases that are so degraded. For that reason, we will use raw isolated data within which outliers are readily identifiable. The idea is to avoid adding mathematical artifacts and blurring information through the development of complex indices. This strategy is an adaptation to the paucity and low reliability of information. Furthermore, attention focuses on metropolitan France because French overseas departments present a very different epidemiological and ecosystem profile, and suffer from a flawed statistical coverage. The number of metropolitan departments entering the analysis is 96.

### Mapping and clustering

Processing will take place in three stages:

- (a) Spatial autocorrelation

At first, with the intent to quantify the spatial relationships between department features, we generated a spatial weights matrix to be used as an input for further statistical developments. Modeling spatial relationships was made using the K-nearest neighbors’ option because of the varying size of feature units (the smallest units -Paris and its periphery- represent only 1 to 2% of the area covered by the largest metropolitan departments) and because of large variations in the spatial distribution of data. The K number of neighbors was fixed to eight. This method is also deemed to be more effective with data values that are not normally distributed, e.g., when known biases exist.

Then we calculated spatial autocorrelation (Global Moran’s I) using the ratio of cumulated data to total population (‰), with the intent to keep global population as a constant reference rather than focusing on infected people or sick patients. This inferential statistic takes as a reference the null hypothesis (randomly distributed attributes) and computes expected and observed index values. From this, the algorithm derives a z-score and p-value for each chosen variable. If the p-value is statistically significant, the null hypothesis is rejected. A positive z-score means that the spatial distribution is more clustered than a random distribution. With a negative z-score, the distribution is more spatially dispersed than would be expected.

These operations are intended to assess the overall pattern and trend of our data.

- (b) Thematic mapping

Secondly, we produced a series of thematic maps illustrating spatial dissemination patterns for the two periods under consideration (lockdown and lockdown lifting). Processed data are those related to cumulated daily new cases, not overall daily cases. The difference between the two lies in contaminated individuals turning negative, the number of which is never recorded. Considering that interdepartmental variations are considerable, we adopted a specific discretization method based on a one-point standard deviation. The number of thresholds increases as statistical heterogeneity grows. Graduated colors represent the ratio of cumulated daily new cases to total population, and graduated symbols the raw number of cumulated new cases. We did not apply any normalization technique to the ratio, to preserve data integrity and avoid removing spatial outliers, but we used the Natural Breaks (Jenks) discretization method to fix the size of symbols. Each specified period has its own symbology parameters, which means that we chose to promote an enhanced synchronic spatial discretization at the expense of diachronic comparison. This was a natural choice, given that each period has its own databases.

To understand the problem more clearly, it should be added that test-related data (purple and orange) are globally underestimated (Fig. 5 and 6). The number of identified positive people is too small to be representative of the population at large and of the overall contamination rate. In France, during the period March 10-May 26, 423 000 tests were conducted, excluding the biological tests carried out in hospitals. Considering that the French population reaches 67 063 000 inhabitants, the overall testing rate over the peak crisis phase is only 6.3‰, with wide interdepartmental variations. Caution should be exercised with these figures since false negative tests might achieve a rate of 10 to 40% depending on the adopted RT-PCR sampling method [34]. Furthermore, there is no way to eliminate multiple counting (people tested many times). Similarly, hospital-related information (blue and red) leave aside the cohort of sick people treated as outpatients or by home care. EHPAD and EMS data do not appear and thematic mapping does not account for the temporal variability of the phenomena studied.

- (c) Data clustering

Given the complexity of analytical result findings, we decided in the final phase to draw up a synthesis of relevant information through the form of data clustering [35]. This venture might be seen as a second assessment of the coronavirus crisis during its most critical phase. In the absence of sufficient data consistency and because of a degraded significance of results, we stated that we would not map the post-lockdown situation as described by the SI-DEP database.

Based on most reliable outcomes, we selected two clustering methods:

1. Hot Spot analysis (Getis-Ord Gi*) addresses spatial variability through the calculation of z-scores and p-values for each department feature [36]. The resulting data allow for the determination of cold and hot spots whose characteristics are determined by constraint parameters. The goal was not here to identify outliers, hence the choice of this algorithm focusing on neighborhood associations. Unlike official mapping, these associations are disconnected from the hierarchy of administrative networks. The conceptualization of spatial relationships is based on fixed distance and Euclidian distance is used. Each feature has at least one neighbor. Hot spot analysis was applied on two variables identified earlier as the most significant inputs (positive tests and new hospital admissions).
2. Multivariate clustering has been chosen to complement the previous analysis and provide for a synthesis without neighboring constraints. This method detects feature similarity using K-Means and optimized seed locations [37]. The algorithm identified eight clusters, but we decided to narrow these results to three groups with the idea to map France on a three alert level basis, as Santé publique France did. Unlike Hot Spot analysis, multivariate clustering does not consider contiguity as a parameter, hence an easier detection of spatial outliers. For this procedure, four input variables were used (tests, positive tests, hospital admissions, and deaths).

SPSS statistics 26 (IBM) and ArcGIS pro 2.5 (ESRI) were used for statistical analysis, thematic mapping, and advanced clustering.

## Results

### Spatial autocorrelation

**Fig. 4:**
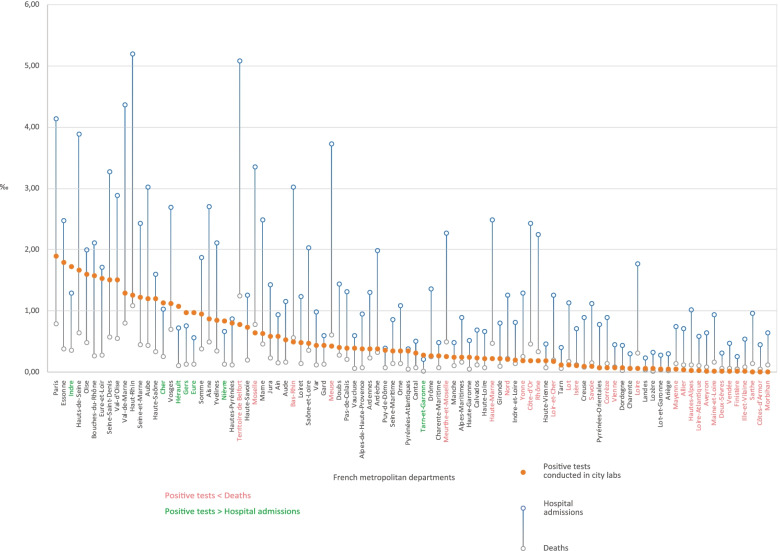
Positive tests, hospital admissions, and deaths by department as a proportion of local population, lockdown phase. Source: Own elaboration based on data from Santé publique France, INSEE. Data sorted out by positive tests conducted in city labs. The variety of local situations is extreme. This chart alone invalidates the “blind” lockdown strategy adopted by the French government. In a limited number of cases (green), the “logical” hierarchy of indicators is respected (positive tests > hospitalizations > deaths). This logic implies that the number of persons tested positive is greater than the number of hospitalized patients, considering that only a small proportion of infected people develop a severe form of the disease. Similarly, the number of hospitalized people should be greater than the number of patients who passed away with/because of SARS-CoV-2, given that mortality is limited within the cohort of hospitalized patients. Most often (black), the overall number of positive tests conducted in city labs is less than the number of hospital admissions (hospitalizations > positive tests > deaths). The position becomes even more curious (red) with positive tests below the level of COVID-19 related deaths (hospitalizations > deaths > positive tests). This situation illustrates the varying reliability of the official database during the emerging phase of the statistical system dedicated to COVID-19, both through time and space. There are several possible reasons for this. (1) The departments registering a small number of COVID-19 cases took more time to implement the statistical platform; (2) Frontline hospitals were already conducting their own tests but these are not reported in the city labs database, which interferes with the results; (3) The reliability and homogeneity of RT-PCR testing procedures might explain certain local discrepancies; (4) The number of reporting public hospital or clinics and city labs has fluctuated over time

Global statistics weighted by the local estimated population offer clear lessons (Table 1). At first, general prevalence remains at a low level. Average positive test rates only reach 0.5‰ during the peak crisis phase and 0.26‰ during the lockdown lifting period. But these are to be interpreted with regard to the low number of tests conducted (maximum of 33 and 52‰ in both periods respectively). We also observe a surprising reversal: during the first phase, the mean hospital admission rate is about three times higher than the positive tests rate, which shows to what extent dissemination has been underestimated. To put it otherwise, the pandemic was already at an advanced stage of development when the first key metrics started to be published. This will have many outcomes in terms of spatial analysis. Identified clusters will not be the initial contamination areas, but further “metastatic” sites. This is confirmed by Fig. 4 on a local basis.

**Table 1:**
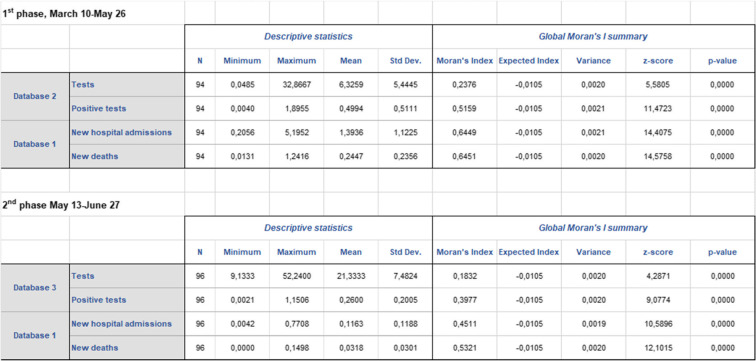
Descriptive statistics and Global Moran’s I summary, ratio of cumulated data to total population (‰)

Source: Own elaboration based on data from Santé publique France

N is the number of spatial units (the departments of metropolitan France). Database 2 (tests conducted in city laboratories) returned a zero information about the two departments of Corsica during the general lockdown phase. This could mean that no tests and no positive cases were reported during the first period. Other options might be considered: no information was transmitted, or Santé publique France found that delivered data were unreliable

Spatial autocorrelation analysis (Global Moran’s I) corroborates the heterogeneity of statistical distributions. With almost null p-values and high z-scores, the estimated likelihood that clustered patterns could be the result of random chance is less than 1%. And this is valid for all four indicators in both phases. Z-scores increase as much as the analysis narrows its focus on the riskiest situations as regards the impact of the virus on health. These figures also show that the significance of further thematic mapping and clustering is extremely high. Slightly lower z-score values in the second phase imply that clustering is almost unaffected by the spread of the virus in statistical terms, e.g. propagation is not associated with impaired clustering effects. The information is counterintuitive because dissemination is often associated with the idea of dilution.

### Mapping the virus spread during the peak coronavirus crisis

**Fig. 5:**
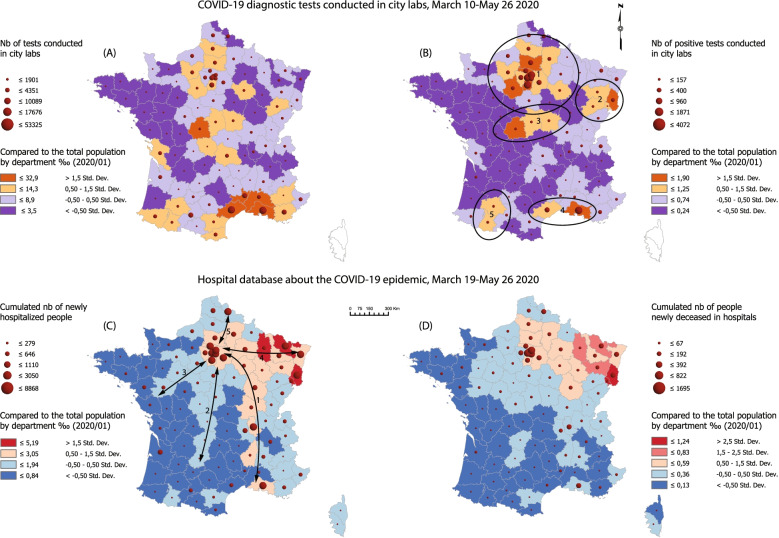
SARS-CoV-2 dissemination patterns by department, March 10-May 26, stringent lockdown period. Source: Own elaboration based on data from Santé publique France, INSEE. This document mirrors screening programs with the treatment of patients in hospitals. From (A) to (D), the entire treatment chain appears implicitly, following the reductionist model described in Fig. 3. The number of people concerned decreases from one map to the next one. The closest approximation to a general prevalence rate is in (B). This map is the most important one because it shows the virus spread in its ‘native’ form, e.g. outside hospital channels. Data collection started at different times for database 1 and database 2. Statistical consistency increases over the first weeks of March as the reporting system gains momentum

**Fig. 6:**
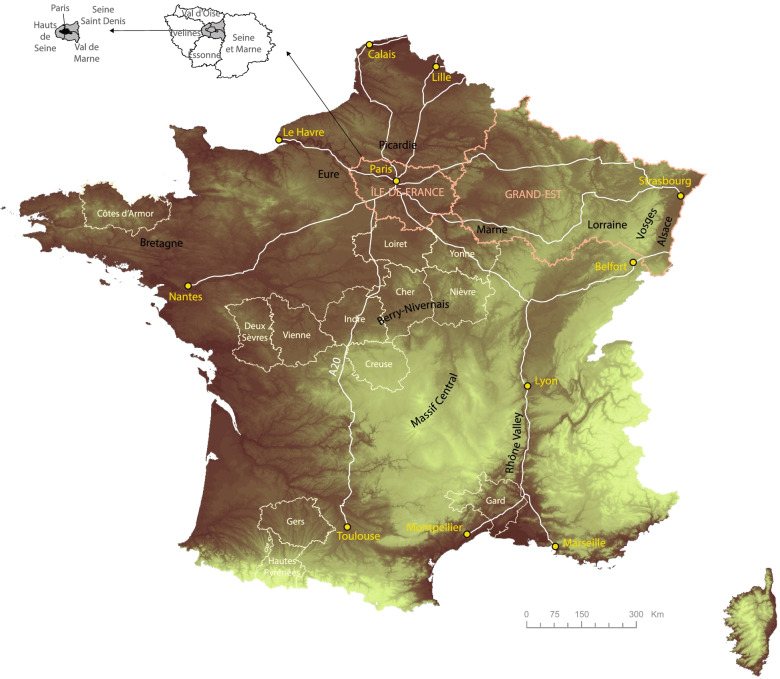
Location map. Source: Own elaboration based on data from NASA SRTM (Shuttle Radar Topography Mission) program, GADM (Database of Global Administrative Areas) 3.6, IGN (French National Geographical Institute). This map serves as a resource for interpreting Figs. 5, 7, and 8, especially through the identification of place names

Figure 5 shows how much the official dashboard derogates to some statistical reality while focusing almost exclusively on ARS databases. This document will be read from (D) to (A) and complemented by Fig. 6 for further geographical names information.

(D) and (C) are quite similar to the vigilance maps provided by the French authorities during the lockdown (Fig. 3). The initial cluster located in Alsace, Lorraine, and around Belfort is clearly identified. However, the Île-de-France appeared at a very early stage as the most important hot spot on a national scale level. This is where the greatest number of deaths has been recognized, with the highest mortality rates observed in the area of Paris, Hauts de Seine and Val de Marne. On May 26, there were 40 088 cumulated new hospital admissions and 7 106 deceased patients in the Île-de-France (38% of the total in France).

Spatial dissemination is part of a twofold logic -zonal contiguity and linear development along traffic routes. Contiguity is an obvious phenomenon insofar as the departments neighboring the initial Alsace cluster are the first ones to be impacted. As the distance to this cluster increases, mortality and hospitalization rates drop more or less steadily.

This areal logic is contradicted or completed by another dissemination process using traffic lanes and adopting a radial pattern centered on Paris:

1. The Rhône Valley is the first development axis, with Lyon and Marseille appearing early as major clusters behind Paris. This southward dissemination was not identified by the authorities. The general death rate is here much lower than the one observed in Northern France.
2. A second axis extends from Paris to South-West France through still highly rural areas along what seems to be the A20 highway.
3. A third axis heading West connects Paris with Nantes.
4. At last, two subsidiary axes link Paris with Lille and Strasbourg. These act in a less direct visible way, because they interact with areas already impacted by the virus on a neighboring-contiguity basis.

Through this, Paris appears to be the main contamination site in France. Observing this crossed axial-areal logic during the lockdown implies that dissemination had already started before March. There is a time lag between the events and their cartographical counter-effects. Particular attention must be paid to the last days before the official lockdown started (March 17, 12 h). The French government announced quarantine enforcement in advance, which resulted in population movements in anticipation of curfew orders. Eleven to 12% of the population living in Paris would have left the capital to settle in a secondary place of residence away from the city [38].

Even more interesting is the analysis of diagnostic tests in (A) and (B). Attention is here focused on the population tested outside hospital units. The geography of positive tests is quite surprising. Five non-neighboring clusters appear:

1. The main cluster around Paris covers six departments, including Eure, Picardie and Marne. The weight of this cluster is overwhelming at a national level: 22 188 positive tests are reported, accounting for 55% of infected people. Furthermore, these results are probably underestimated because Paris and its outskirts have conducted considerably fewer diagnostic tests than other provincial departments.
2. The primary Eastern contamination cluster is much more limited spatially. The perimeter only covers four departments with 1 716 positive cases (4% of the total). The small size of these figures coupled with the saturation observed in the hospital units in the Grand-Est and with the high local death rate raises many questions that would require deeper consideration. Did local hospitals suffer from a major shortage of equipment? Do we have to blame specific genetic profiles or did treatment protocols prove to be inappropriate?
3. A third rural cluster covers the departments of Indre, Cher, and Nièvre.
4. In Southern France, the situation is quite different. With statistics provided at a department level, spatially limited clusters are less visible. The Rhône Valley fades away and two local contamination clusters emerge in Marseille and Montpellier, plus another grouping in the Gers and Hautes-Pyrénées departments. These early contamination areas were given little attention. The disease pattern differs from the Northern profile: the infection gives rise to a lower number of hospital admissions and a reduced number of deaths.

To sum up, we identified three changing cluster patterns:

-In the North, corresponding to the Île-de-France and Vosges: limited number of diagnostic tests, massive hospital admissions, and high death rates.
-In the South, centered on local urban clusters: enhanced screening strategy, limited hospital admissions, and low death rate.
-The Pyrenean and Berry-Nivernais clusters have a pattern quite similar to the previous one, with a “controlled” epidemic dissemination avoiding the saturation of hospital capacities. However, these develop in rural areas and their connectivity within the national transportation grid is more limited.

### Mapping dissemination during the lockdown lifting period

**Fig. 7:**
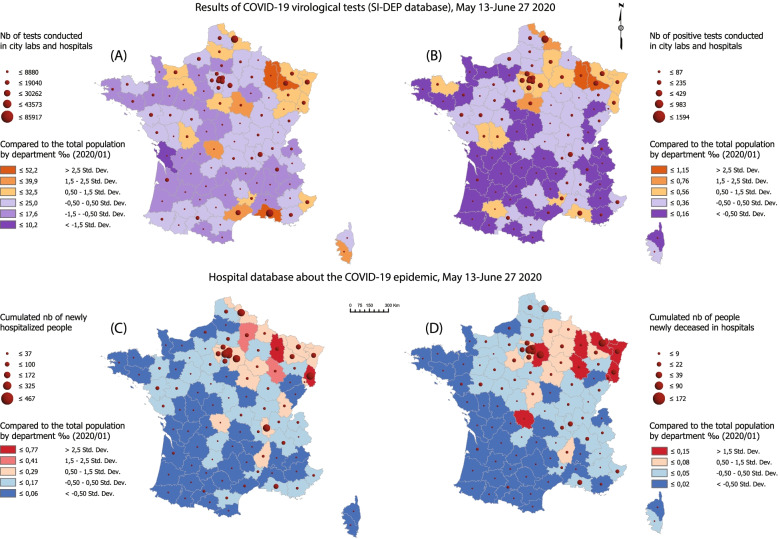
SARS-CoV-2 dissemination patterns by department, May 13-June 27, lockdown lifting period. Source: Own elaboration based on data from Santé publique France, INSEE

Figure 7 illustrates the post-lockdown situation up to the beginning of the summer break. The creation of the SI-DEP database does not allow anymore differentiation between community and hospital testing centers, hence a strong correlation between (A), (B), (C), and (D). This new strategy for statistical data management necessarily strengthens the power of the hospital system. Information is globally blurred and less significant. The geographical pattern identified before seems to settle down. The number of tests increases, positive results fall as well as the overall number of hospital admissions and deaths. All indicators turn green, although various warning signs start to emerge suggesting that the epidemic continues to spread silently. Many unexpected outliers arise: Côtes-d’Armor, Deux-Sèvres and Vienne, Gard, Loiret and Yonne, Creuse are departments contiguous with the previously identified clusters, which means that areal dissemination is still ongoing. This evolution heralds further summer developments with the virus spread reaching the country’s remotest areas.

More than four months after the COVID-19 crisis started in France, despite the global decline of the epidemic, the Grand-Est and Île-de-France areas remain active. The linear pattern of the virus spread seems to carry less weight, but this is probably an outcome of two simultaneous phenomena: local singularities emerge; and the epidemic goes into recession in various clusters the size of which was limited.

### Clustering approach

**Fig. 8:**
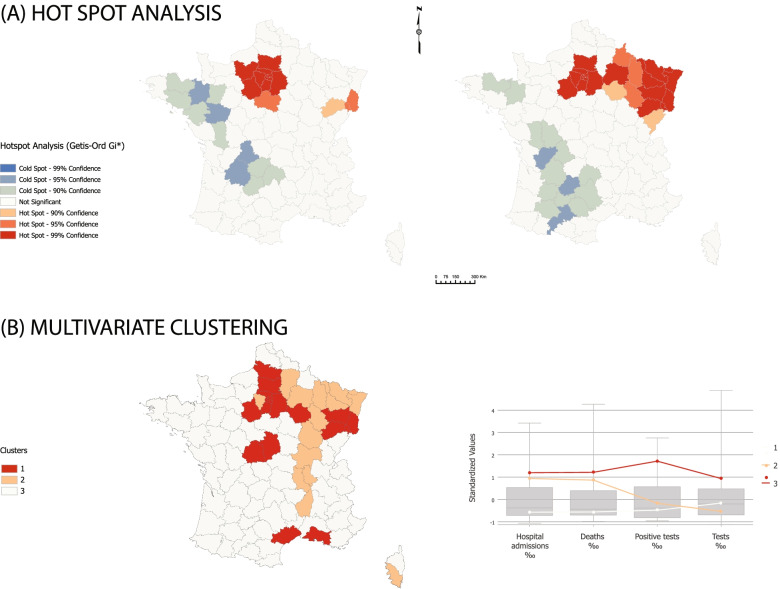
Hot Spot analysis and Multivariate clustering, lockdown phase. Source: Own elaboration based on data from Santé publique France, INSEE

Figure 8 produces results diverging once again significantly from governmental vigilance maps. In (A), the hot spot analysis identifies two positive and two negative clusters, confirming that geographical patterns addressing positive tests (prevalence) differ markedly from hospital indicators. Parisian Basin and Alsace are top-infected areas, which is in line with thematic mapping results. The western part of Massif Central and Bretagne are the most preserved areas. New hospital admissions associate Grand-Est and Île de France within the same cluster. The North-East/South-West gradient is a classical geographical division within the country. In both cases, the Paris-Lille and Paris-Marseille axes are not identified because of an enhanced number of local outliers without contiguity.

Multivariate clustering produces interesting, much more accurate results because of the missing neighborhood constraint. This time, the COVID-19 geography is funnel-shaped with Île-de-France and Grand-Est forming the upper cone and the Rhône Valley being the lower tube. Three outlying departments complete the spatial system: two southern urban clusters in Marseille and Montpellier, and another rural cluster in the Berry region. The graph in (B) confirms that these groupings result mainly from a clearly differentiated statistical answer of positive tests.

## Discussion

We observed significant discrepancies between the official prospects on the virus spread in France and our results. Many infected sites remained unseen despite the existence of dedicated observatories [39]. This paradox leads us to believe that further investigations would greatly benefit from accurate ancillary databases focusing on community medicine, provided that access rights be granted in the future [40]. Despite an impressive number of academic publications, the geography of SARS-CoV-2 is still in the early stages of research development.

This work offers a distinct advantage: the replicability of the method is allowed by the availability of the algorithms used. Given the uncertain reliability of data sources, we chose not to revert to complex analysis techniques that would have “compensated” for obvious defects of the official databases. The objective was to remain as close to the initial “signals” as possible. Derived indicators and optimization-normalization techniques were bypassed to avoid the elimination of statistical-spatial outliers, which is crucial for detecting hot spots. The overall replicability of the exercise is however conditional on minimum quality standards: “over-derived” or “over-agglomerated” statistics lead to a loss of integrity, which is why the experiment was not prolonged during the post-lockdown phase.

One of the most problematic issues about COVID-19 management by the authorities in France -but also in other places worldwide- is a limited capacity to identify hot spots. This is mainly due to structural reasons:

- 1-Metrics are related to territorial administration and spatially-defined health care systems. As a result, statistical production interferes with the real geography of hot spots (border effects). This problem might be solved by an enhanced accuracy of spatial databases and/or through a more systematic use of mapping solutions based on kriging and spatial interpolation techniques.
- 2-Official COVID-19 databases report on the status of the health care system rather than improve our insight into the coronavirus itself, both in demographic or spatial terms. A systematic watch system is still to be invented. These ambiguities appeared clearly during data quality analysis, and we assume that this probably caused further interpretation problems, which in turn led to distorted decisions as regards the management of the epidemic by the authorities. Many narratives based on incidence or prevalence rates miss the point, avoiding to mention that a significant proportion of the population may carry the pathogen without symptoms, with or without transmission capacity, and that a good portion of infected people chose to remain away from health services. Other systematic surveillance tools are to be developed outside the area of hospitals and community medicine with the purpose to bring to the fore background or hidden inputs (the invisible people listed in Fig. 2). Wastewater observation might be an appropriate solution, although the spatial coverage allowed by this system is limited to communities equipped with sewerage networks, which leaves aside the majority of rural municipalities as well as many peri-urban areas [41].
- 3-Other issues are aggregation and scale dependency: the higher the statistical/spatial aggregation level, the lower the significance of results. This is especially true when environmental variables are introduced in modelling exercises. Reintroducing specific (not generic) indicators at a local scale might allow for a better understanding of the dynamics of recurring or emerging hot spots. Since October 21 2020, Santé publique France publishes a short list of statistical indicators at an IRIS sub-departmental level: incidence rate, testing rate, and positivity rate [42]. IRIS are “aggregated units for statistical information” and serve as standard units for the dissemination of infra-municipal data by the INSEE. To ensure anonymity, no absolute values are provided. Although the initiative seems to be a step in the right direction towards a more effective micro-scale approach, the chosen indicators suffer from the same faulty assumptions. Furthermore, because the postal code of patients/tested people could not allow for the identification of the corresponding IRIS code, the geographic distribution of people was calculated using a probabilistic algorithm (25% of the cases). With the same causes producing the same effects, the availability of these data did not really provide the knowledge basis necessary to improve the action policies of the government.

This work demonstrates how much the legal and spatial management of quarantine results from partially biased political choices. During the general lockdown 35 departments never moved from the green alert level to orange or red. One third of the country stayed in complete lockdown without any other reason than safety precaution or missing data able to establish that local communities had been spared by contamination. Unfortunately, there is no possible analysis of the spread within major cities, which leaves open the question of how and why urban districts were unequally affected by the disease.

Apparently, the general lockdown did not stop the virus spread probably because dissemination had already reached an advanced stage before the decision was made. It simply delayed contamination. The government would have chosen to slow down the progression of COVID-19 and reduce emergency department overcrowding, perhaps waiting for the hypothetical development of herd immunity. If the assumption is correct, spatial epidemiology was a secondary issue [4]. The resolution of the crisis is thus expected to emerge from background processes affecting households and small communities where contaminated individuals inevitably continue to proliferate quietly, away from the hustle and bustle of media platforms and from the frenetic activity of emergency services. Lockdowns are scale transfers.

The official statistical system severely constrains and restricts opportunities for approaching complex mutating phenomena at detailed scale. At the end of June, after about six months spent under COVID-19 pressure, only 2.9% of the metropolitan population had been tested. With 60 000 identified positive cases from March 10 to June 27, 0.09% of the French population had been officially infected by the virus. In the absence of mass testing and considering that the rate of positive serology results is much higher than RT-PCR [43], these figures are of limited reach but they are the only kind of data available at the moment.

This absence reveals a political crisis behind the epidemiological crisis: local communities were completely marginalized during the epidemic. Despite official positions supporting decentralization as a beneficial political process over the last decades, communes and federations of municipalities have shown the extent of their powerlessness. Similarly, community and city medicine were sidelined for the benefit of hospital units directly placed under the supervision of the ARS and the prefects.

French Jacobinism/centralism emerges strengthened from the events. Paris being by far the main SARS-CoV-2 cluster in France as early as March, the question is raised as to whether the country could uphold a stringent lockdown of its capital against the preservation of economic activity and free movement in the rest of the country. The French government decided otherwise. The precautionary principle has fulfilled its role as a catalyst for crisis management and centrality reinforcement. This is an obvious defeat of applied spatial epidemiology.

## Conclusions

This work demonstrates how much health care policies could manage epidemic crises on a geographical basis much more effectively, using relatively simple techniques that would in the end prevent the reproduction of “blind” lockdown decisions. To reach that goal, however, public attention should deviate partially from the hospital, patient-based “paradigm” and refocus on upstream, community or environmental-based detection initiatives. Early cluster detection is a key input for boosting the benefits of anti-contagion policies, and applied spatial epidemiology plays an essential role in it.

The study validates different works focusing on the geographic spread of emerging pandemics [44], especially as regards the impact of spatial heterogeneity and neighborhood relationships in determining the extent and pace of dissemination [44]. We found early neighborhood-zonal and distant-linear dissemination patterns based on transportation networks. Obviously, mobility and connectivity are of critical importance [45, 46], to such a point that spatial integration, travel frequency, and eventually network density -rather than population density- might be seen as factors positively associated with COVID-19 propagation [47]. This probably falls within the worldwide context of an enhanced connectedness between places, otherwise called “space–time convergence” [8]. The phenomenon produces dissemination patterns sometimes assimilated with “metastatic” growth [48].

At this stage, we propose a scenario where contamination takes its source in specific peripheral places operating with international passenger transport services; as a second step, central urban places are infected and become core-contamination areas; then, through a boomerang effect, dissemination moves back towards peripheral places without leaving any area unaffected, depending on the degree of state centralization and inter-connectedness. Less integrated countries or areas would present “natural” defense mechanisms and barriers. The scenario is replicable at different scales and time shifting.

We also note that infection patterns and local mortality are clearly decorrelated both in space and time. The reasons for this are not fully established, and further investigation is required. For instance, this work would greatly benefit from additional analyses using phylogenetic and environmental landscape data [49]. Besides, little information exists on the very controversial issue of differentiated treatment protocols adopted within and outside hospital units.

Methodological issues are at stake in addressing the current crisis. Not surprisingly, our results diverge from other studies that use general, or “blind”, socio-economic and environmental variables to explain the extent of the spread [16]. We found that a number of classical methods in the area of epidemiology are fairly inappropriate in addressing spatial dynamics. For instance, standardization and normalization are known useful techniques widely used in health sciences. However, these might produce reverse effects when applied to datasets elaborated by territorial administrations whose purpose is precisely to publish normalized metrics -with varying degrees of success. The extraordinary complexity of living beings, societies, and environments do not easily comply with administrative requirements. Simulation is apparently a panacea for advanced epidemiological studies, and this is sustained by strong political demand, but these approaches have shown their limits in terms of predictability. This is why cluster modeling might benefit from the advances achieved by complex sciences and chaos systems, which give a prominent place to statistical/spatial outliers, self-reproductive and non-linear mechanisms.

Further research developments are now required to address high-scale dissemination issues, without which no clear understanding of the pandemic might be reached. Given that the spread follows a highly clustered pattern regardless of the stage of infection, priority must be granted to the analysis of existing or emerging clusters [50]. We need to characterize them at a sufficient level of detail, identify geographical dynamics and self-consistency, leaving aside the areas marginally affected by the virus.

This work opens up new research avenues, among which are the following: (1) The detection of SARS-CoV-2 RNA in sewerage systems is a promising way to investigate intra-urban dissemination channels; (2) Superspreading events were identified as the riskiest situations in terms of infection potential, but identifying and mapping the most vulnerable places as regards cross-contamination could also be useful for the determination of by-passing strategies; (3) In organizational terms, studying the “spontaneous” response of decentralized, local health care systems might be interesting to assess the efficiency of community-based organizations in fighting the epidemic. Most of these initiatives imply changing the scale of analysis, reintroducing space-territory within the loop, and looking away from hospitals and emergency services. 

## Data Availability

The datasets supporting the conclusions of this article are freely available online. -Hospital database related to the COVID-19 epidemic, http://data.gouv.fr/en/datasets/donnees-hospitalieres-relatives-a-lepidemie-de-covid-19/. -Database related to COVID-19 tests conducted in city, http://data.gouv.fr/en/datasets/donnees-relatives-aux-tests-de-depistage-de-covid-19-realises-en-laboratoire-de-ville/. -Database related to the results of COVID-19 virological tests SI-DEP, http://data.gouv.fr/en/datasets/donnees-relatives-aux-resultats-des-tests-virologiques-covid-19/.
